# Gaseous Elemental Mercury and Total and Leached Mercury in Building Materials from the Former Hg-Mining Area of Abbadia San Salvatore (Central Italy)

**DOI:** 10.3390/ijerph14040425

**Published:** 2017-04-15

**Authors:** Orlando Vaselli, Barbara Nisi, Daniele Rappuoli, Jacopo Cabassi, Franco Tassi

**Affiliations:** 1 Department of Earth Sciences, Via G. La Pira, 4-50121 Florence, Italy; franco.tassi@unifi.it; 2 CNR—Institute of Geosciences and Earth Resources, Via G. La Pira, 4–50121 Florence, Italy; jacopo.cabassi@unifi.it; 3 CNR—Institute of Geosciences and Earth Resources, Via Moruzzi, 1–56124 Pisa, Italy; b.nisi@igg.cnr.it; 4 Unione dei Comuni Amiata-Val D’Orcia, Via del Colombaio, 98-53023 Gallina, Castiglion d’Orcia, Siena, Italy; d.rappuoli@uc-amiatavaldorcia.si.it

**Keywords:** gaseous elemental mercury, Hg-mining areas, Abbadia San Salvatore, Central Italy, total and leached mercury, building material, remediation

## Abstract

Mercury has a strong environmental impact since both its organic and inorganic forms are toxic, and it represents a pollutant of global concern. Liquid Hg is highly volatile and can be released during natural and anthropogenic processes in the hydrosphere, biosphere and atmosphere. In this study, the distribution of Gaseous Elemental Mercury (GEM) and the total and leached mercury concentrations on paint, plaster, roof tiles, concrete, metals, dust and wood structures were determined in the main buildings and structures of the former Hg-mining area of Abbadia San Salvatore (Siena, Central Italy). The mining complex (divided into seven units) covers a surface of about 65 ha and contains mining structures and managers’ and workers’ buildings. Nine surveys of GEM measurements were carried out from July 2011 to August 2015 for the buildings and structures located in Units 2, 3 and 6, the latter being the area where liquid mercury was produced. Measurements were also performed in February, April, July, September and December 2016 in the edifices and mining structures of Unit 6. GEM concentrations showed a strong variability in time and space mostly depending on ambient temperature and the operational activities that were carried out in each building. The Unit 2 surveys carried out in the hotter period (from June to September) showed GEM concentrations up to 27,500 ng·m^−3^, while in Unit 6, they were on average much higher, and occasionally, they saturated the GEM measurement device (>50,000 ng·m^−3^). Concentrations of total (in mg·kg^−1^) and leached (in μg·L^−1^) mercury measured in different building materials (up to 46,580 mg·kg^−1^ and 4470 mg·L^−1^, respectively) were highly variable, being related to the edifice or mining structure from which they were collected. The results obtained in this study are of relevant interest for operational cleanings to be carried out during reclamation activities.

## 1. Introduction

Total Gaseous Mercury (TGM) refers to the sum of Gaseous Elemental Mercury (GEM), Gaseous Oxidized Mercury (GOM) and Particulate Bounded Mercury (PBM), e.g., [1], the latter two being usually indicated as RM (Reactive Mercury; e.g., [2]). GEM (or Hg^0^) is by far the most abundant form of Hg in the atmosphere (>95%) since it has high stability and volatility and low solubility with a residence time between 0.6 and two years [3,4]. On the contrary, GOM and PBM (defined by Hg^+2^ compounds that consist of mercuric halides, mercuric sulfate, mercuric nitrite and mercuric hydroxide [5]) are removed in a relatively short time, i.e., days or weeks [6].

According to [7], TGM emitted from anthropogenic activities to the atmosphere is about three times higher than that emitted by natural sources. Coal combustion, waste incineration and cement production are the most important TGM anthropogenic sources (about 2200 Mg·y^−1^; e.g., [8,9]), whereas those related to natural emissions are mainly due to volcanic and hydrothermal systems (up to 830 Mg·y^−1^; e.g., [10,11,12,13]). Recently, [14] computed that TGM contributions from both natural (primary emissions + re-emissions) and man-made sources are equal to 7527 Mg·y^−1^.

The U.S. Government Agency for Toxic Substances and Disease Registry has ranked mercury as the third most toxic substance on the planet after arsenic and lead [15,16], and it is distributed in the hydrological, pedological and atmospheric geochemical spheres. Mercury affects cellular, cardiovascular, hematological, pulmonary, renal, immunological, neurological, endocrine, reproductive and embryonic systems of humans, e.g., [17]. According to [18], atmospheric mercury poses two specific risks: (i) a direct one, which involves the inhalation of gaseous mercury, causing different problems to human physiology, e.g., [18,19], and (ii) a collateral one, which refers to the transformation of the Hg-species, i.e., from either GEM into GOM or GOM into methyl-Hg, the latter being the most toxic form of mercury, e.g., [20,21,22,23,24]. Many international projects have provided detailed information on the distribution of GEM on a global scale, e.g., [25,26]. Moreover, actions to ban the opening of new Hg-mines, the closure of those already existing and the elimination of any Hg-bearing products from daily life are presently undertaken (The United Nation’s Minamata Convention [27]).

Serious health problems are caused by human exposure to inorganic mercury during the exploitation of ore containing mercury, especially when Hg-bearing rocks are roasted to produce Hg°, e.g., [28,29,30,31,32,33,34,35], or other occupational activities where mercury is used, e.g., [36,37,38,39].

In this paper, we present new original data on the spatial and temporal distribution of GEM in the main buildings and structures of the former Hg-mining area of Abbadia San Salvatore (Siena, Central Italy) and total and leached concentrations of mercury determined on different building materials in order to: (i) assess to what extent GEM contamination occurs; (ii) characterize the amount of mercury deposited and adsorbed in the building materials of the most important edifices (including the furnace-bearing structures); and (iii) provide indications for minimizing the impact to the workers who are about to initiate the first phase of remediation. The main actions will consist of the removal of paint, plaster, roof tiles and dust. Operational activities are also expected to occur in the structures that are still hosting the Gould and Nesa furnaces, where the highest concentrations of GEM were recorded [40].

## 2. The Study Site

**Figure 1:**
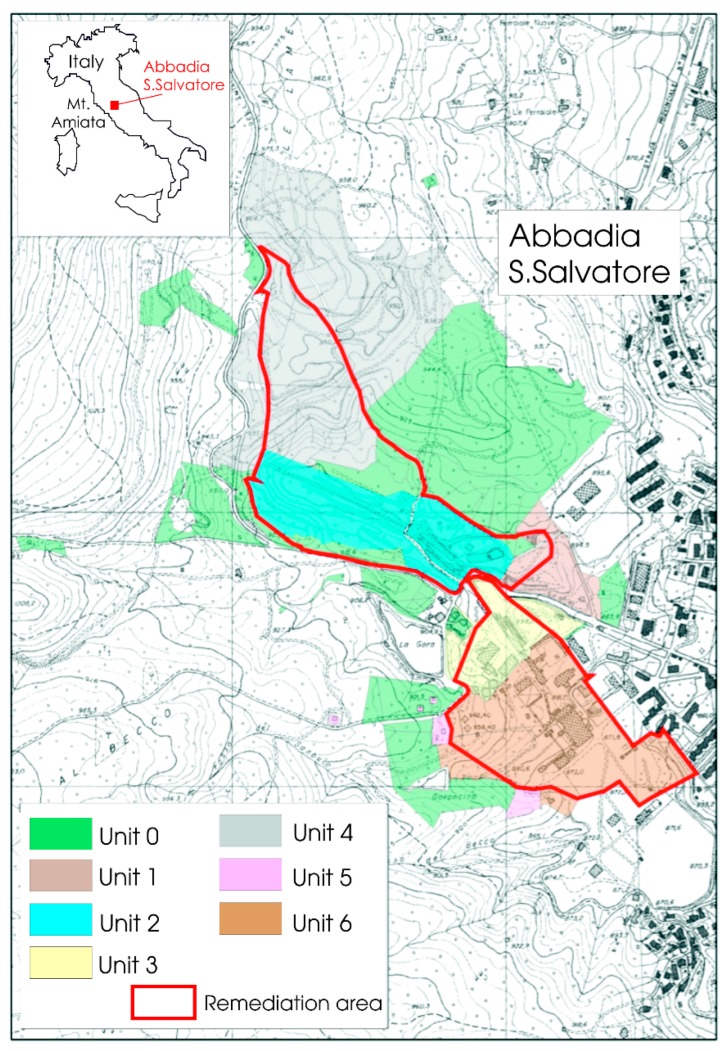
Location of the Hg-mining district of Abbadia San Salvatore (Central Italy) and subdivision into seven units according to the expected different concentrations of mercury present as both GEM and total and leached mercury in the building materials of the edifices hosted in the former mining area.

The world-class Hg-mining district of Abbadia San Salvatore is located in Southern Tuscany (Central Italy; Figure 1), and it is related to the volcanic activity of the Mt. Amiata silicic complex [41,42], whose products, mainly consisting of trachytic to olivine latitic lava flows and domes, were emplaced between 305 and 231 ka [43,44].

The very first exploration studies at Abbadia San Salvatore date back to 1846. Mercury production started in 1899 when the Cermak Spirek furnaces were ignited for the first time. The old mining area also included a large deposit of wood for the furnaces, some driers and a small water pool that was used to cool down the gaseous mercury as it was passing through the condensers. In the following years, horizontal (Gould) and vertical (Nesa) furnaces, new dryers and transportation belt systems and slug deposits were installed. The production activity at Abbadia San Salvatore terminated in 1976, since the exploitation of mercury was not economically sustainable, and the use of mercury declined due to its noxious and toxic effects. In 2008, an agreement between the Municipality of Abbadia San Salvatore and the former owner of the mining concession (E.N.I., National Agency for Hydrocarbons, AGIP Division) was signed to transfer the ownership of the reclamation to the public institution. In the agreement, remediation actions were addressed to an environmental rehabilitation of the mining areas and buildings for museum purposes and public greens [40,45]. It was estimated that more than 100,000 tons of liquid mercury were produced during the activity of the Abbadia San Salvatore mining district [46,47], whilst about 10% of the total production was released as Hg fumes into the atmosphere [45,48].

After the closure of the mining activity, E.N.I.-AGIP Division produced numerous documents where operational activities to remediate the Hg extraction and processing areas were reported, although cessation of the mining activities, which occurred without a scheduled basis, left the decontamination issue open. In fact, liquid mercury and tailing mounds are still occurring in the study area.

In 1998, the Tuscany regional authorities (Regional Decree No. 1447) produced specific guidelines (named “Norma Amiata”) for the remediation of the metallurgic activity related to the Hg-mining production areas. The most important points were, as follows: (1) outdoor and indoor concentrations of GEM have to be <300 and <500 ng·m^−3^, respectively; and (2) concentrations of Hg in leached soils, terrain and building materials have to be <1 μg·L^−1^ after leaching with CO_2_-saturated water.

**Figure 2:**
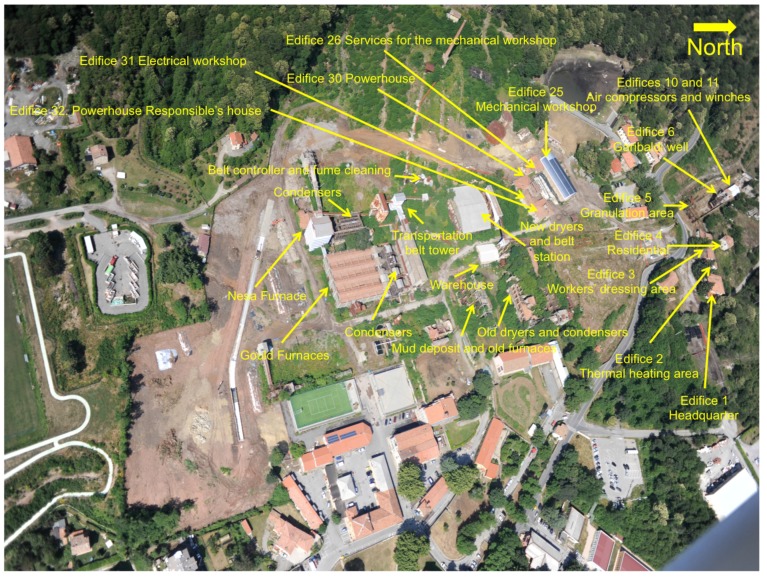
Photos from an ultralight vehicle of the main edifices and mining structures from Units 2, 3 and 6 (see Figure 1) with a description of their use when the mining district was active.

On the whole, the mining complex has a surface of about 65 ha and contains mining structures and managers’ and workers’ buildings (Figure 2). Previous studies, e.g., [32,40,47,49], evidenced the relatively high concentrations, though heterogeneously distributed, of GEM and total mercury in the mining structures and related building materials, respectively. Accordingly, the Municipality of Abbadia San Salvatore divided the mining complex area into seven different units (Figure 1) [50], Unit 6 containing the most heavily contaminated structures (Figure 2) [40], as follows.

Unit 0: This sector is dominated by large green areas mainly consisting of chestnut trees and Mediterranean scrub and located far from any mining structure. No remediation actions are expected [49].

Unit 1: It is located in the eastern entrance of the mining site, and only a small portion is included in the remediation area. No Hg contamination was recorded, being situated far from the sites where liquid mercury was produced [49].

Unit 2: It includes several edifices, such as the mining headquarter building, the workers’ dressing room and showers and mining structures, e.g., grounding area, mineral conveyor belts, the Garibaldi well.

Unit 3: It consists of several edifices, among which are: the electrical cabin, the mechanical workshops and an old edifice where furnaces, dryers and condensers were present.

Unit 4: This area (named “Le Lame”) is located to the north of the mining area where most mining wastes were accumulated.

Unit 5: It is the smallest unit and hosts the armory and the guardian’s house. No Hg contamination was recorded [49,50].

Unit 6: It is situated to the south of the former mining area close to the urban center of Abbadia San Salvatore. The Gould and Nesa furnaces, condensers and dryers and the main material storing areas are located in this unit.

In this paper, we focused our attention on those edifices and mining structures belonging to Units 2, 3 and 6, which urge a prompt remediation, being characterized by the highest concentrations of mercury [40,49]. GEM measurements and chemical analyses of man-made materials were carried out in the edifices indicated in Figure 2.

## 3. Materials and Methods

**Figure 3:**
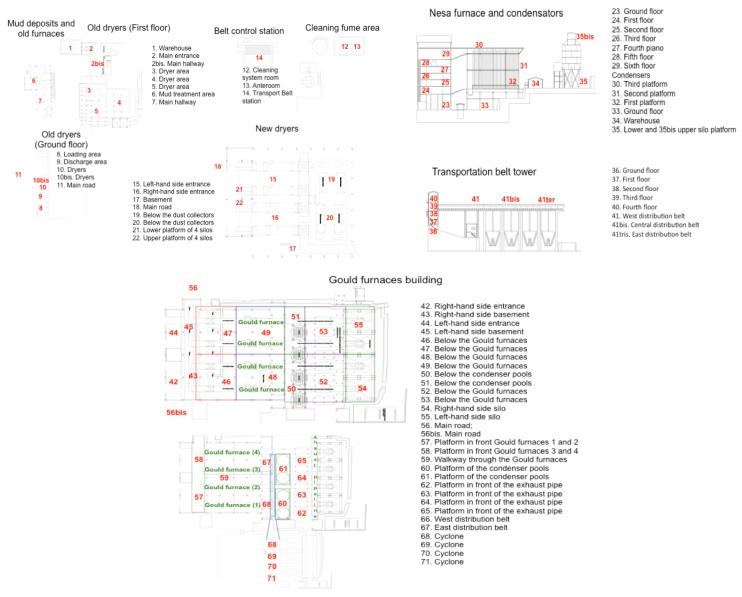
Edifices and mining structures hosted in Unit 6 (Figure 1 and Figure 2) and the location of the 77 spots (and relative description) where GEM surveys by Lumex 915+ were carried out in February, April, July, September and December 2016. GEM data and mean, minimum and maximum temperatures during the surveys are in Supplementary Material S2.

Nine surveys of GEM measurements were carried out from July 2011 to August 2015 for those buildings and structures located in Units 2, 3 and 6 (Figure 1 and Figure 2). In addition, GEM measurements were also performed in February, April, July, September and December 2016 in 77 selected spots inside and outside edifices and mining structures of Unit 6 (Figure 3).

Real-time GEM measurements in air were carried out using a portable Lumex (915+) analyzer. This device is based on Zeeman Atomic Absorption Spectrometry using High Frequency Modulation of Light Polarization (ZAAS-HFM; [51]). Application of Zeeman background correction and a multipath analytical cell provides high selectivity and sensitivity. The accuracy of the method is 20% [52]. The detection limit is governed by shot noise and equals CaDL (Characteristic Absolute Detection Limit) = 2 ng·m^−3^ (average measuring time = 5 s) and CaDL = 0.3 ng·m^−3^ (average measuring time = 30 s) at a flow rate of 20 L min^−1^ for GEM determination in ambient air and industrial and natural gases. The dynamic range covers four orders of magnitude (2–50,000 ng·m^−3^).

The GEM portable instrument was maintained at a height of 150 cm from the ground, while the operator was slowly moving around each room. Each measurement consisted of the acquisition of the GEM data every one second and calculating the mean values every 30, 60, 90, 120, 150 and 180 s. The GEM data were thus reported as the mean value calculated after 180 s of measurements. During the in-door measurements, the recorded data for each room were varying by ±10%. A similar procedure was adopted when measuring GEM outside of most edifices and structures and carried out at a distance of about 1 m from the walls.

Mean, minimum and maximum temperatures were obtained by two meteorological stations located at about two hundred meters from the former Hg-mining district and available at [53,54].

All of the samples for the analysis of total and leached mercury were collected by using gloves; to remove paint, plaster, rust, concrete and wood, a hammer, chisel and spatula, cleaned with HCl and acetone, were used. Two soil samples, collected at about 10 m from the edifice containing the Gould furnaces (Figure 2), were dried at room temperature and then sieved at 2 mm. The <2-mm fraction was used for the determination of total and leached mercury.

Paint, plaster, roof tiles, dust and wood were collected from Units 2, 3 and 6 and analyzed at the Laboratories of Gruppo CSA Ricerche (Rimini) by DMA (Direct Mercury Analyzer)-80, according to the procedure reported in [55]. All samples were ground and homogenized. According to the expected Hg concentrations, a few tens to hundreds of milligrams of each sample (analyzed in triplicate) were weighed in a sample boat, thermically decomposed in an oxygen flow at 650 °C and transferred to a Mn_3_O_4_-CaO catalyst, which removed possible interference substances, e.g., halogens and molecular nitrogen and sulfur oxides. The Hg°-rich vapors were interacting with an Au-amalgamator that acted as a selective trap for mercury. Then, mercury was promptly released by increasing the temperature up to 900 °C and transferred by the O_2_ flow to the measurement system that consisted of atomic absorption. Absorbance was measured at 253.65 nm, obtained by an interferential filter that acted on the radiation emitted by an Hg cold vapor lamp at low pressure. A calibration curve was built with appropriate Hg° standards. The analytical error was <10%.

Metallic material (e.g., furniture and rust) and the two soils were digested with aqua regia according to the method UNI EN 13657:2004 at the Laboratories of Gruppo CSA Ricerche (Rimini) and analyzed by ICP-AES (Agilent 720ES) following the recommendations reported in UNI EN ISO 11885:2009. The analytical error was <10%.

Leaching tests consisted of weighing about 10 g of fine-grained material into a 100-mL beaker to which 50 mL of CO_2_-saturated MilliQ water were added. CO_2_-saturated MilliQ water was obtained by bubbling pure CO_2_ into a Pyrex^®^ bubbler, which was previously cleaned with ultrapure HCl (1:1), for 15 min until a pH of 4.5 was reached. The suspension was periodically swirled for about 3 h and allowed to decant overnight. The supernatant was filtered at 0.45 μm with cellulose nitrate filters. Mercury was then analyzed by ICP-AES at the Laboratories of Gruppo CSA Ricerche (Rimini).

## 4. Results

### 4.1. Hg° Measurements in the Main Edifices and Mining Structures

The GEM data measured in the nine surveys carried out from July 2011 to August 2015 in the edifices and structures of the Abbadia San Salvatore mining district and belonging to Units 2, 3 and 6 are reported in Supplementary Material S1 along with the respective planimetry and mean, minimum and maximum temperatures when the GEM measurements were carried out, whilst those related to the same period and those determined (Figure 3) in February, April, July, September and December 2016 in Unit 6 are listed in Supplementary Material S2. Gaseous mercury background values for Mt. Amiata are 3–5 ng·m^−3^ [40], while in the urban area of Abbadia San Salvatore, the recorded values were <10 ng·m^−3^ [56].

Remarkable variations were observed during the GEM surveys, mostly related to seasonal variations. It is worth mentioning that doors and windows from edifices from Units 2 and 3 had been closed for many years after the closure of the mining activity. They contained old furniture, metallic spare parts, wood, rock samples, and so forth. Later on, these materials were removed and analyzed for total and leached Hg before their disposal (see below). Consequently, the air quality of most rooms was improved. For the sake of clarity, below, we summarize the most relevant results obtained during the nine (Units 2 and 3) and fourteen (Unit 6) GEM surveys, whilst the full set of data, including mean, minimum and maximum temperatures, are reported in Supplementary Materials S1 and S2.

Edifices belonging to Unit 2 and the respective GEM concentrations (in ng·m^−3^) were, as follows:

Edifice 1 (headquarters): It consists of five and 14 rooms located at the ground and first floor, respectively. GEM concentrations were from 20 to 182 ng·m^−3^ (ground floor) and from 5 to 602 ng·m^−3^ (first floor). GEM measurements carried out along the perimeter of the edifice were between 8 and 56 ng·m^−3^.

Edifice 2 (thermal heating area): It has one room that showed GEM values always <50 ng·m^−3^.

**Figure 4:**
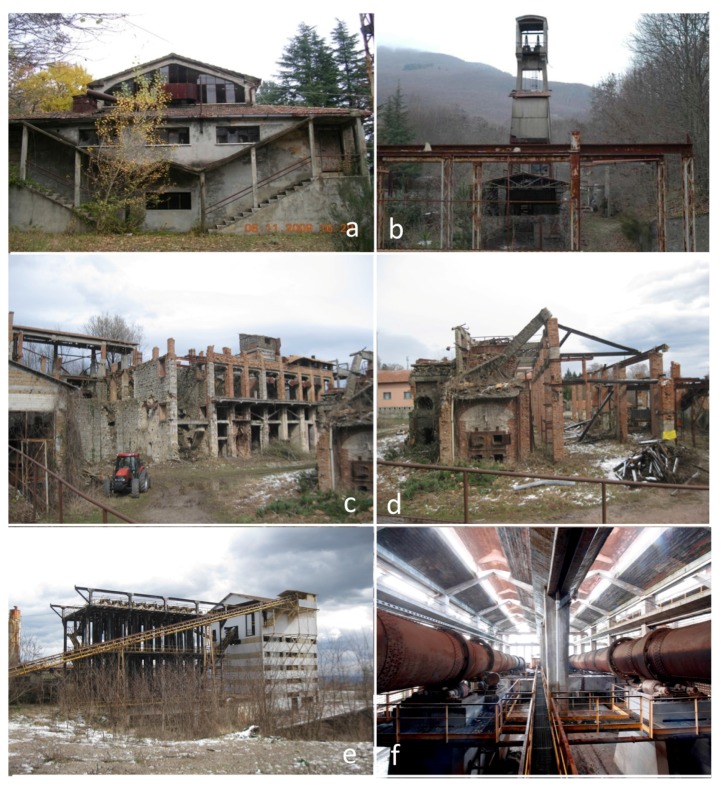
Buildings and mining structures analyzed in this work: (**a**) miners’ dressing building; (**b**) Garibaldi well; (**c**) old driers and condensers; (**d**) old mud deposit and furnaces; (**e**) Nesa furnace and condensers; (**f**) building hosting the Gould furnaces.

Edifice 3 (workers dressing building, Figure 4a): It is formed by three floors: the ground and first floors and a mezzanine, the latter being almost completely destroyed since the roof partly collapsed. The ground floor has six rooms where GEM reached values up to 932 ng·m^−3^, although in September 2014, 1686 ng·m^−3^ were measured in Room A (Supplementary Material S1). In the first floor, GEM values were <144 ng·m^−3^, while in the mezzanine, they were up to 113 ng·m^−3^.

Edifice 4 (residential): It consists of two floors. This building is seriously damaged, and consequently, GEM measurements (up to 111 ng·m^−3^) were carried out only sporadically due to possible collapses.

Edifice 5 (granulation area): It is characterized by a ground floor and three basements; in July 2011, the highest GEM values were recorded in the basement floors: 1100, 1250 and 13,600 ng·m^−3^, respectively. GEM values at the ground floor never exceeded 280 ng m^−3^.

Edifice 6 (Garibaldi well; Figure 4b): GEM measurements were carried out close to the main entrance of the well, now closed for safety reason, with values ≤267 ng.m^−3^.

Edifices 10 and 11 (air compressor and winch areas, respectively): They contain power supply machines to run the elevator of the Garibaldi well. GEM values were <100 ng.m^−3^, whilst along the perimeter of the building, they were <66 ng·m^−3^. In 2015, before the collapse of the roof, GEM values up to 85 ng·m^−3^ were measured in the westernmost room.

The edifices belonging to Unit 3 and the respective GEM (in ng.m^−3^) values were, as follows:

Edifice 25 (mechanical workshop): It has a surface of about 1000 m^2^ and hosts a mezzanine and three small rooms (about 10 m^2^ each). GEM measurements were carried out in three distinct sectors of the building (Supplementary Material S1), where the highest concentrations were up to 3608 (close to the main entrance), 3968 (in the middle of the building) and 2131 (close to the rear entrance) ng·m^−3^. In the mezzanine, GEM values up to 2350 ng m^−3^ were recorded. Eventually, GEM concentrations in the three rooms were <2350 ng·m^−3^, whilst 368 ng.m^−3^ were measured along the perimeter of the building.

Edifice 26 (services for the workers of the mechanical workshop and pigment production area): It is divided into nine sectors where GEM concentrations up to 4453 ng·m^−3^ were measured. Perimetral values were up to 497 ng m^−3^.

Edifice 30 (powerhouse): It consists of two floors where relatively variable GEM concentrations were measured and comprised between 950 and 58 ng·m^−3^.

Edifice 31 (electrical workshop): It has four rooms, and the GEM concentrations were up 1551 ng·m^−3^.

Edifice 32 (house of the supervisor in charge of the powerhouse): It has a small basement (about 15 m^2^) and two floors. GEM values were relatively high and mostly varied between 333 and 2358 ng·m^−3^ (ground floor) and 168 and 6896 ng·m^−3^ (first floor).

Unit 6 contains the main structures that were used to produce liquid mercury, and GEM concentrations were measured with two different approaches. From July 2011–August 2016, Lumex measurements were carried out in order to recognize where the highest GEM values were located. Then, from February–December 2016, 77 sites situated in different edifices were systematically and repeatedly measured. A description of each measured site is reported in Figure 3. The complete set of data related to gaseous mercury is fully listed in Supplementary Material S2.

Old dryers and condensers, mud deposits and old furnaces (Figure 2; points marked from 1–11 in Figure 3 and Supplementary Material S2): These old buildings partly collapsed (Figure 4c,d). GEM concentrations were highly variable in terms of space and time. The highest values were measured at Points 6 (2480, up to ng·m^−3^), 9 (up to 3660 ng.m^−3^) and 10bis (up to 1133 ng·m^−3^).

Main conveyor belt station and cleaning fume area (Figure 2; points marked from 12–14 in Figure 3 and Supplementary Material S2): GEM contents were >200 ng·m^−3^; the highest concentrations was 1630 ng·m^−3^.

New dryers (Figure 2; points marked from 17–22 in Figure 3 and Supplementary Material S2): GEM concentrations were spatially and temporally highly variable, since, for example, at Points 16 and 17, they were spanning from 47–6606 and from 161–4910 ng·m^−3^, whereas the lowest values (up to 625 ng·m^−3^) were measured at Points 15, 16, 21 and 22, the latter two being located in the conveyor belt area.

The Nesa furnace, condensers, silo platforms and warehouse (Figure 2; points marked from 23–35bis in Figure 3 and Supplementary Material S2): The Nesa furnace is hosted in an about 30-m high edifice (Figure 4e). It was built in the 1960s and was functioning for a very short time due to stability problems. Some mining material is still present in both the furnace and silo. GEM measurements in the platforms of the silo were only performed in 2016. With the exception of the GEM measurements carried out in the upper platforms of the condensers (Points 30 and 31), the other sites were characterized by values >1000 ng·m^−3^ at least during one of the surveys. The highest GEM concentrations (up to 10,096 ng·m^−3^) were measured at Points 32–35.

Belt transportation tower (Figure 2; points marked from 36–41ter in Figure 3 and Supplementary Material S2): This building consists of five floors and a >50-m long horizontal conveyor belt, which was divided into three parts for this study. GEM concentrations were highly variable and ranged from 57–3192 ng·m^−3^, with the exception of Point 37, where a value of 10,835 ng·m^−3^ was measured in September 2016.

Gould furnaces building (Figure 2; points marked from 42–71 in Figure 3 and Supplementary Material S2): This is the edifice where liquid mercury was produced and consists of four Gould furnaces (Figure 4f), condensers, condensation pools, cyclones to force the fumes from the furnaces into the condensers, an exhaust pipeline, several silos used for the storage of the mining material before roasting and conveyor belts. In this area, liquid mercury is still condensing, and occasionally, small liquid mercury pools are observed [45]. As expected, this building is to be regarded as the most contaminated site among all of the edifices and mining structures of the former mining area of Abbadia Sal Salvatore. In some of the investigated sites, GEM concentrations were >50,000 ng·m^−3^. More than 200 points (Supplementary Material S2) were measured during the 14 surveys, and in almost 90% of them, GEM concentrations were >1100 ng·m^−3^; >60% were >3000 ng·m^−3^; and >30% were >10,000 ng·m^−3^.

### 4.2. Total and Leached Mercury

Concentrations of total (in mg·kg^−1^) and leached (in μg·L^−1^) mercury from different building materials and rock fragments still present in the transportation belts are listed in Table 1. Total mercury showed for the same type of material highly variable values depending on the edifice or mining structure from which it was collected. Roof tiles were characterized by the lowest total Hg contents, being comprised between 0.8 and 17.5 mg·kg^−1^ for those edifices located relatively far from the liquid mercury production area, whereas those collected in the mining structure hosting the old driers and Gould furnaces, total Hg concentrations were of 36.2 and 485 mg·kg^−1^, respectively, both showing relatively high contents of leached Hg (0.2 and 485 μg·L^−1^, respectively). We remark that roof tile samples collected from the building hosting the Gould furnaces showed a relatively low concentration of total Hg (4.1 mg·kg^−1^), although leached Hg was >1 μg·L^−1^. Concrete samples also showed a relatively wide concentration range for both total and leached mercury (from 2.2–46,580 mg·kg^−1^ and <0.1 and 4,470 μg·L^−1^), the highest values being related to concrete samples collected from the building hosting the Nesa and Gould furnaces.

Total Hg concentrations in ordinary and tuff bricks ranged from 4.8–11,535 mg·kg^−1^, while leached Hg was between 0.3 and 2250 μg·L^−1^, the highest values being found in the mining structures of Unit 6. Notice that tuff bricks showed, on average, a higher content of leached Hg, being relatively more porous than ordinary bricks. Both total and leached Hg values measured in drilled cores of various tuff bricks from Edifice 25 (Figure 2) were systematically higher than those measured at the brick surface (Table 1). Similarly, paint was characterized by higher concentrations of total and leached mercury (from 5.5–281 mg·kg^−1^ and from <0.1–37.6 μg·L^−1^, respectively) when compared to the underlying plaster (from 10.8–708 mg·kg^−1^ and from <0.1–392 μg·L^−1^, respectively). Unfortunately, where the highest concentrations of total mercury in plaster were found, paint was scanty or even absent.

All of the abandoned machineries and instrumentations were partially covered by rust; thus, several samples from different edifices and mining structures were also analyzed for total and leached mercury, the former varying from 1.3 (railing rust, Edifice 3) to 3390 (rust from the Nesa furnace) mg·kg^−1^. Leached mercury from rust collected from the Nesa and Gould furnaces was measured only on two samples (351 and 717 μg·L^−1^, respectively).

Three dust samples from the old and new driers were analyzed for total and leached mercury with values up to 13,680 mg·kg^−1^ and 1020 μg·L^−1^, respectively.

Mineral wool from the Nesa furnace had total mercury of 420 mg·kg^−1^, whereas leached mercury was 833 μg·L^−1^.

Wood pylons, wooden beams and tables showed concentrations of total mercury up to 57.2 mg·kg^−1^. The wooden beam from the edifice hosting mud deposit and old furnaces had leached mercury of 0.4 μg·L^−1^.

Condensers connected to the Nesa and Gould furnaces are made of crystalline isotactic polymer (Moplen^®^). Significant differences in terms of total and leached mercury were recorded since fragments from the condensers of the Gould furnaces showed higher concentrations than those related to the Nesa furnace: 3020 mg·kg^−1^ and 660 μg·L^−1^ and 420 mg·kg^−1^ and 1.1 μg·L^−1^, respectively.

As previously mentioned, most (metal and wood) furniture, rock samples and wooden and roofs (the latter related to collapsed parts of some buildings) were removed from Edifices 1, 2 and 3 and stored outside and piled up. Wood and metal furniture and wooden beams and roofs were analyzed for total and leached mercury by collecting fragments from each accumulation, which consisted of about 1–2 tons each (Table 1). The highest total mercury contents were measured in the wooden beams (34.6–907 mg·kg^−1^), while the lowest ones were found in the metal furniture (2.5–6.7 mg·kg^−1^), although the latter had relatively high leached mercury (0.7–6.1 μg·L^−1^).

Eventually, two fine powdered samples from the ore deposit, still present in the transporting belts, one lime sample located in the bottling area of liquid mercury and two soil samples, collected outside of the building hosting the Gould furnaces, were also analyzed (Table 1). No leached mercury was measured in the powdered rock samples. It is worthwhile to mention that in the two soils, waste products (e.g., calcine, bricks, roof tiles, and so forth) were present [45]. High mercury concentrations were recorded in the powdered samples (up to 10,800 mg·kg^−1^) and the soils (up to 73,670 mg·kg^−1^), the latter also showing high contents of leached mercury (up to 6640 μg·L^−1^). The lime sample had values of total and leached mercury of 181 mg·kg^−1^ and 105 μg·L^−1^, respectively.

## 5. Discussion

### 5.1. Spatial and Temporal Distribution of GEM

Mercury vapor is absorbed through inhalation, e.g., [57,58]; it bonds to S-bearing amino acids and can reach the brain, e.g., [59]. Prolonged exposure to mercury vapor may induce neurological dysfunction, and even low-level exposures are reported to produce weakness, anorexia, weight loss, and so forth [60]. Changes in personality, loss of memory, depression and occasionally delirium were reported as some of the symptoms when humans are exposed to high levels of mercury vapor [61]. The work in [32] evidenced that miners from Abbadia San Salvatore were less exposed to Hg° since no native mercury was present in the ore deposit. Differently, workers involved in the smelting process, cinnabar pigment production, soot purification, laboratory work and bottling showed high concentrations of mercury in their blood and urine. The high GEM values measured in this study for those buildings where native mercury was produced and treated can thus affect the operational activity of the operators during remediation processes.

**Figure 5:**
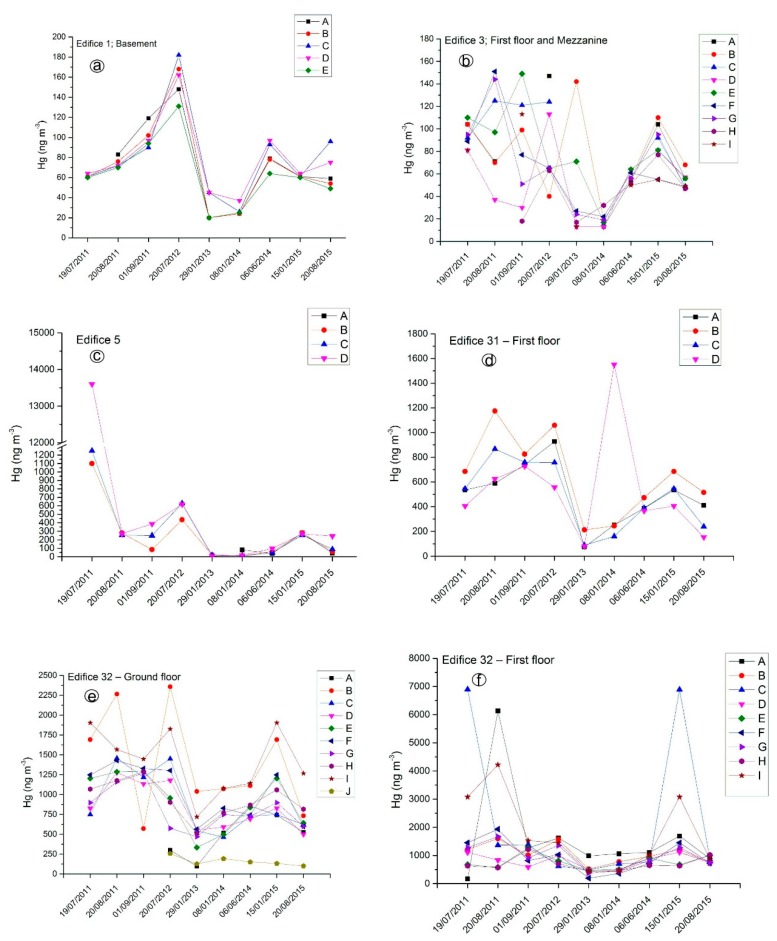
Gaseous Elemental Mercury (GEM) concentrations (in ng·m^−3^) versus time (from July 2011–August 2015) for selected edifices located in Units 2 and 3; (**a**) Edifice 1: Basement; (**b**) Edifice 3: First floor and Mezzanine; (**c**) Edifice 5; (**d**) Edifice 31: First floor; (**e**) Edifice 32: Ground floor; (**f**) Edifice 32: First floor. The full set of GEM data is reported in Supplementary Material S1.

GEM concentrations in the edifices and mining structures from the former Hg-mining area of Abbadia San Salvatore showed a strong variability in time and space (Supplementary Materials S1 and S2 and Table 1), mostly depending on ambient temperature and operational activities carried out in each building, respectively. To better evidence such differences, time variations (from July 2011–August 2015) for selected buildings located in Units 2 and 3 are reported in Figure 5. Surveys carried out in the hotter period (from June–September) showed the highest GEM values, although in Edifice 1 and Edifice 3, the limit defined by the Tuscany Region (500 ng·m^−3^) was never exceeded. Values well above 500 ng·m^−3^ were recorded in the edifices where machinery used for the extraction of mercury is still present (Edifice 5) or where workers and miners were operating (Edifice 31 and Edifice 32). Moreover, the mercury production area of Unit 6 is relatively close to these edifices (Figure 2). The high GEM concentrations measured in the buildings hosting the Gould and Nesa furnaces can be displaced to Edifices 31 and 32 when wind is blowing from the south [40], increasing the GEM contents during wintertime, as occasionally recorded (Figure 5).

**Figure 6:**
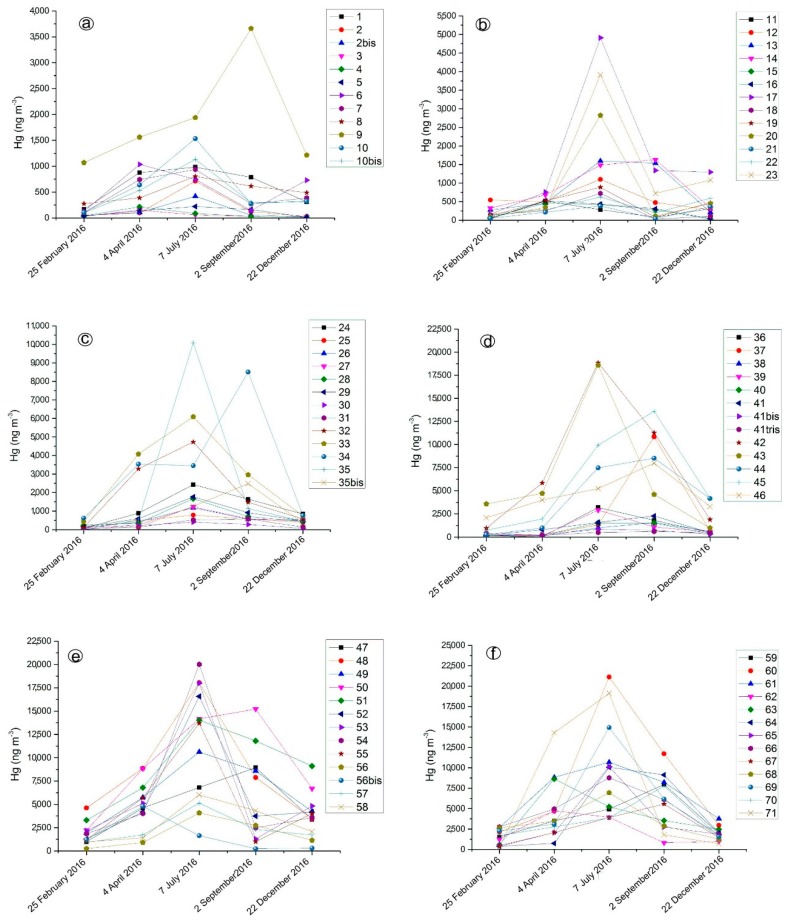
GEM concentrations (in ng·m^−3^) versus time (from February–December 2016) for selected edifices and mining structures located in Unit 6. The numbering reported in each figure corresponds to that reported in Figure 3. The full set of GEM data is reported in Supplementary Material S2.

In Unit 6, GEM concentrations (Supplementary Material S2) were much higher than those recorded in the edifices of Units 2 and 3 (Supplementary Material S1), being able to saturate the GEM measurement device (>50,000 ng·m^−3^). The spatial and temporal GEM variability in Unit 6 is shown by separately considering the five surveys carried out in 2016 (Supplementary Material S2), during which 77 spots were systematically analyzed in February (mean, min and max temperatures: 6.2, 3.0 and 8.0 °C, respectively), April (mean, min and max temperatures: 14.7, 10.5 and 17.1 °C, respectively), July (mean, min and max temperatures: 22.0, 15.4 and 27.2 °C, respectively), September (mean, min and max temperatures: 20.8, 13.7 and 25.7 °C, respectively) and December (mean, min and max temperatures: 5.6, −0.1 and 10.8 °C, respectively). As shown in Figure 6, in summertime, i.e., July 2016, GEM had the highest concentrations. Occasionally, relatively high concentrations were also detected in September. During the colder months, GEM concentrations strongly decreased, sporadically dropping down to <500 ng·m^−3^. It is worth mentioning that even those edifices that had partly collapsed (e.g., buildings hosting mud deposits, old furnaces and old driers), hence being more affected by meteorological events with respect to the partly closed structures (e.g., the mining structures hosting the Nesa and Gould furnaces), showed GEM concentrations >500 ng·m^−3^.

The most important finding is that GEM concentrations almost systematically were higher than the in-door threshold of 500 ng·m^−3^, although they were much lower than those recorded by [40] in 1982, when the mining activity shut down. GEM values up to 250,000 ng·m^−3^ were indeed measured.

### 5.2. Total and Leached Mercury Concentrations in the Building and Stored Materials

**Figure 7:**
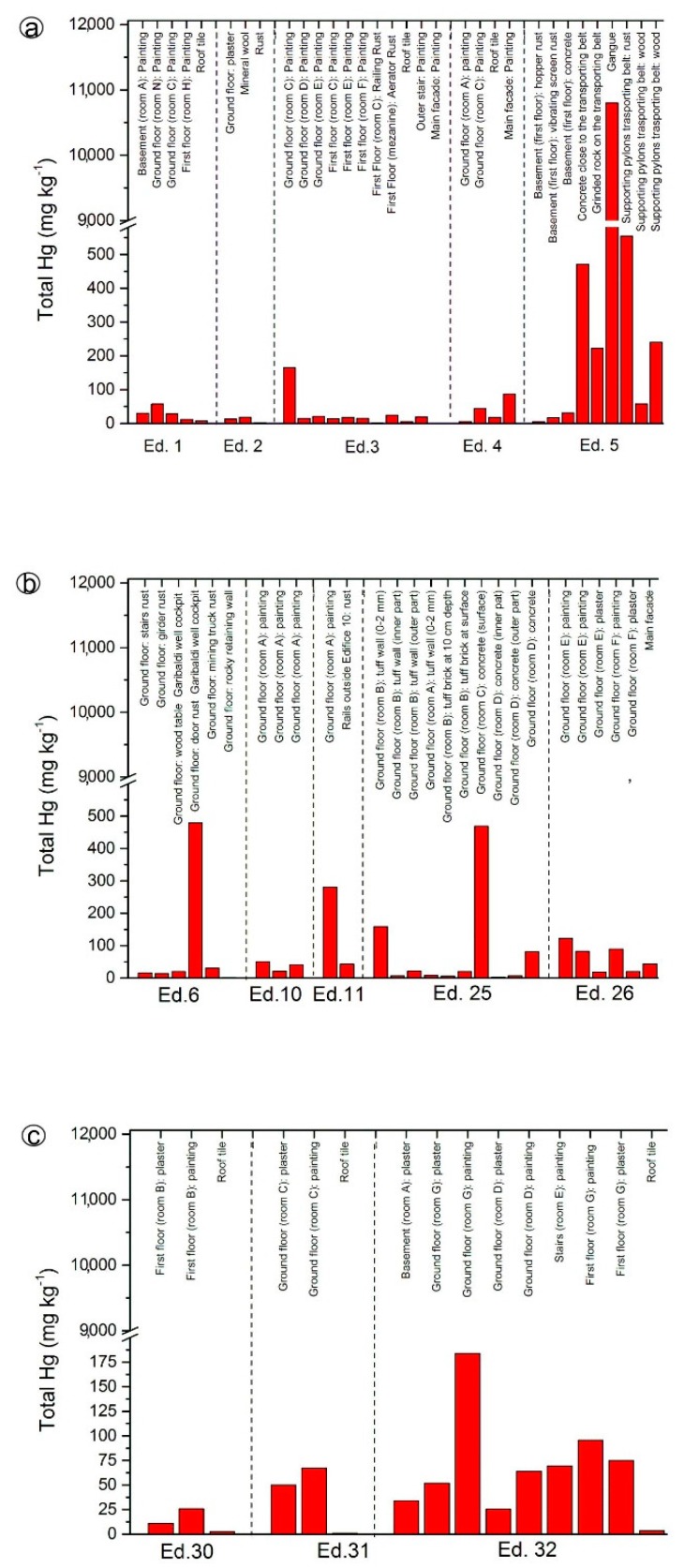
Bar diagrams for total Hg concentrations (in mg·kg^−1^) measured in different building materials from Units 2 (from Ed. 1 to Ed. 11) and 3 (from Ed. 25 to Ed. 26). Ed.: Edifice.

**Figure 8:**
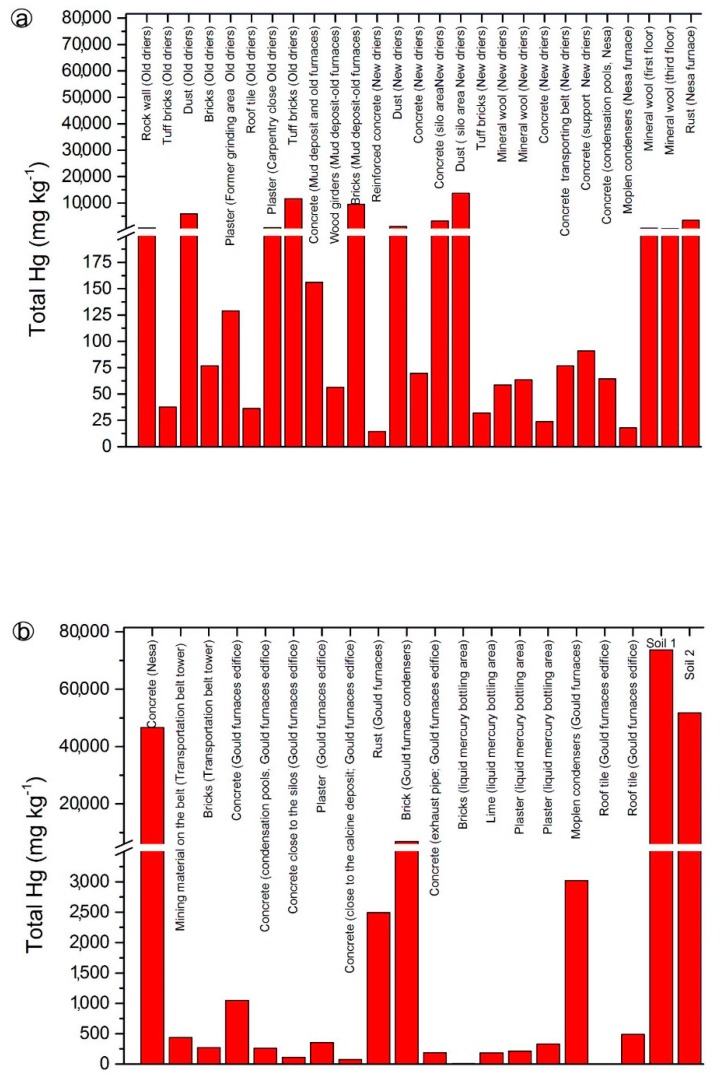
Bar diagrams for total Hg concentrations (in mg·kg^−1^) measured in different building materials from Unit 6; (**a**) samples from old driers, mud deposit and old furnaces, new driers and Nesa furnace, (**b**) samples from Nesa and Gould furnaces, liquid mercury bottling area and soils.

To the best of our knowledge, no reference total and leached mercury concentrations are available for building materials exposed to mercury contamination, and consequently, a comparison between unaffected and contaminated concrete, wood furniture, rust, dust, and so forth, is not presently viable. We remind that the highest concentration admitted of leached mercury for the disposal of any kind of material in ordinary landfill is 1 μg·L^−1^. We also remind that: (i) >100 ktons of liquid mercury were produced, and 10 ktons were lost in the atmosphere; (ii) the buildings hosting the Nesa and Gould furnaces, the condensers and the old and new driers (Figure 2) are important GEM emitters (Supplementary Material S2); and (iii) after the closure of the mining activity, GEM concentrations were much higher [49] than those measured in this study. Thus, concentrations of total and leached mercury mostly represent the amount of mercury absorbed during the mining and post-mining activity. The spatial distribution of total mercury in the analyzed material collected from the edifices of the former mining area (Figure 7 and Figure 8) showed increasing concentrations approaching Unit 6 (Figure 2, Supplementary Material S1 and Table 1). As previously evidenced, paints are more enriched in mercury than plaster underneath, suggesting that mercury absorbed at the surface only partly diffuses inside the analyzed wall. Tuff bricks, concrete and rust also appeared to be efficient mercury absorbers (Figure 7).

Setting aside the materials from Edifice 5, those analyzed from Unit 6 showed total mercury concentrations of one order of magnitude higher than those recorded in the edifices from Units 2 and 3, independently of the type of analyzed material. Concrete, paint, mineral wool and crystalline isotactic polymers of the condensers showed the highest concentrations of total mercury, likely because they were exposed (and still they are) for a longer time to GEM-rich fumes.

We evidenced the persistence of gaseous mercury despite the fact that the mining activity at Abbadia San Salvatore terminated in 1976, although high GEM concentrations were also measured in other decommissioned mining districts (e.g., Almaden, Spain, and Idrija, Slovenia; [18]) or even in apartments and studios built in Hoboken (NJ, USA), where a tool-and-dye company and, successively, a factory of manufacturing mercury valor lamps operated in the past [62,63]. GEM concentrations >1000 ng·m^−3^ were recorded [64]. Unfortunately, no data on paint and plaster are available, but according to our findings, it is matter of fact that building materials are good absorbers of mercury.

Studies on Hg mobility have been carried out in order to proceed with soil-remediation techniques in mining areas, e.g., see [65] and the references therein. The work in [66] assessed that adsorption/desorption processes control the behavior of Hg in the soil, suggesting that mercury can occur in dissolved, non-specifically and/or specifically adsorbed, chelated or precipitated forms. The work in [67] evidenced that Hg mobility depends on its chemical speciation, which can be dictated by soil parameters, including pH and redox potential [62,68], and their interactions. In addition, Hg transformations operated by microbial activity, via methylation and demethylation processes (likely not applicable to building materials investigated in this paper), may further mobilize or stabilize mercury, e.g., see [69] and the references therein.

**Figure 9:**
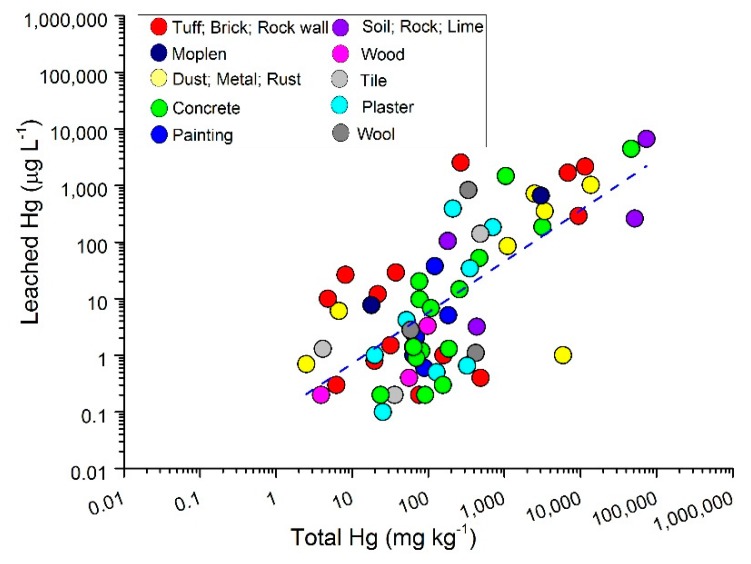
Binary diagram of total (in mg·kg^−1^) versus leached mercury (in µg·L^−1^) for different materials collected from building and mining structures of Units 2, 3 and 6 and grouped according to their characteristics.

In the absence of specific investigations aimed to understand how Hg is speciated, a binary diagram of total (in mg·kg^−1^) versus leached mercury (in μg·L^−1^) is reported in Figure 9. The analyzed materials were grouped according to their characteristics. A positive correlation (Pearson’s r = 0.7) between total and leached mercury is observed, i.e., the higher the total mercury, the higher its removal by leaching. With the exception of the two soil samples collected outside the building hosting the Gould furnaces, the exposure to gaseous Hg favors the increment of mercury in the building materials.

The equation of the straight line depicted by total (THg) versus leached (LHg) mercury (Figure 9) is:

LHg = −1.0472 + 0.90177 * THg(1)

Assuming that no leachable mercury is expected to occur in such material, we may speculate that the value of 1.16 mg·kg^−1^ might be considered a sort of reference concentration for uncontaminated building materials. The THg/LHg ratio is relatively variable and comprised between 105 and 588,0000, suggesting that at high total Hg concentrations, leached Hg is relatively high (Figure 9), although as a percentage, the latter represents a small fraction. This may indicate that most Hg is present in a stable form, and leaching by water-saturated CO_2_ is able to remove a minimal quantity of Hg, though often higher than the limit defined by the Norma Amiata (1 μg·L^−1^).

## 6. Conclusions

GEM concentrations in the edifices and mining structures from the former Hg-mining area of Abbadia San Salvatore (Tuscany, Central Italy) showed in most cases concentrations >500 ng·m^−3^. The highest values were recorded in summer and dramatically decreased in winter when the ambient temperatures were approaching 0 °C. High concentrations of total and leached mercury were also detected in the building materials (e.g., tuff bricks, roof tiles, concrete), suggesting that they act as Hg-absorbers when affected by high GEM concentrations.

The reclamation project in the former mining area of Abbadia San Salvatore is still at the beginning, and it has not yet involved the buildings of the workers and miners, nor the liquid mercury production areas. Thus, the results obtained in this study are of relevant interest for the operational cleanings to be carried out during the reclamation activities. Operators are expected to dress in appropriate personal protective equipment and use machinery (e.g., hydro-blasters) to avoid the dispersion of mercury in the environment during the removal of paint, plaster, dust, rust, and so forth. This is highly recommended for both the operators’ safety and that of the inhabitants living nearby, the urban center of Abbadia San Salvatore bordering the former mining area (Figure 1). To better monitor the operational activities, continuous acquisition of GEM data is suggested, and samples of urine, blood and hair for mercury concentrations should be collected in statistically-significant populations of operators prior to and after the reclamation, since several months will likely be necessary to complete the cleaning activity, particularly in the most contaminated sites.

## Figures and Tables

**Table 1:** Concentrations of total (mg·kg^−1^) and leached (μg·L^−1^) mercury analyzed in different materials collected from the former mining area of Abbadia San Salvatore; n.d. not determined.

SAMPLING SITE	Total Hg	Leached Hg	SAMPLING SITE	Total Hg	Leached Hg
	mg·kg^−1^	µg·L^−1^		mg·kg^−1^	µg·L^−1^
EDIFICE 1			EDIFICE 31		
Basement (room A): Painting	29.6	n.d.	Ground floor (room C): Plaster	50.0	<0.1
Ground floor (room C): Painting	28.3	<0.1	Ground floor (room C): Painting	67.0	2.2
Ground floor (room N): Painting	57.1	<0.1	Roof tile	0.8	<0.1
First floor (room H): Painting	11.5	<0.1	EDIFICE 32		
Roof tile	6.8	n.d.	Basement (room A): plaster	34.0	<0.1
EDIFICE 2			Ground floor (room G): plaster	51.9	4.2
Ground floor: plaster	12.8	<0.1	Ground floor (room G): painting	184	5.1
Mineral wool	18.5	n.d.	Ground floor (room D): plaster	25.4	0.1
Rust	2.2	n.d.	Ground floor (room D): painting	63.8	1.0
EDIFICE 3			Stairs (room E): painting	69.3	2.1
Ground floor (room C): Painting	165	<0.1	First floor (room G): painting	95.6	<0.1
Ground floor (room D): Painting	14.3	n.d.	First floor (room G): plaster	74.9	<0.1
Ground floor (room E): Painting	20.5	n.d.	Roof tile	3.3	<0.1
First floor (room C): Painting	13.8	<0.1	MATERIAL REMOVED AND DISPOSED FROM EDIFICE 1 AND 3		
First floor (room E): Painting	17.8	<0.1	Wood girders 1	1.9	<0.1
First floor (room F): Painting	15.3	<0.1	Wood girders 2	25.6	<0.1
First Floor (room C): Railing Rust	1.3	n.d.	Wood girders 3	0.8	<0.1
First Floor (mezzanine): Aerator Rust	24.3	n.d.	Wood girders 4	8.4	<0.1
Roof tile	6.0	n.d.	Wood furniture 1	3.9	0.2
Outer stair: Painting	19.0	<0.1	Wood furniture 2	9.17	<0.1
Main facade: Painting	n.d.	<0.1	Metal furniture 1	2.5	0.7
EDIFICE 4			Metal furniture 2	6.7	6.1
Ground floor (room A): Painting	5.5	n.d.	Wood roof 1	98.5	3.3
Ground floor (room C): Painting	44.0	n.d.	Wood roof 2	907	<0.1
Roof tile	17.5	n.d.	Wood roof 3	34.6	<0.1
Main facade: Painting	86.5	<0.1	Wood roof 4	176	<0.1
EDIFICE 5			UNIT 6		
Basement (first floor): Hopper Rust	4.8	n.d.	Rock wall (Old driers)	486	0.4
Basement (first floor): Vibrating Screen Rust	16.0	n.d.	Tuff bricks (Old driers)	37.6	29.2
Basement (first floor): Concrete	31.0	<0.1	Dust (Old driers)	5880	1.0
Concrete close to the conveyor belt	471	<0.1	Bricks (Old driers)	76.6	0.2
Grinded rock on the conveyor belt	222	n.d.	Plaster (Former grinding area in the Old driers)	129	0.5
Gangue	10,800	n.d.	Roof tile (Old driers)	36.2	0.2
Supporting pylons of the conveyor belt: Rust	554	n.d.	Plaster (Carpentry close to the Old driers)	708	183
Supporting pylons of the conveyor belt: Wood	57.2	n.d.	Tuff bricks (water depuration area close to the Old driers)	11,535	2160
Supporting pylons of the conveyor belt: Wood	240	n.d.	Concrete (Mud deposit and old furnaces)	156	0.3
EDIFICE 6			Wood girders (Mud deposit and old furnaces)	56.3	0.4
Ground floor: Stairs Rust	15.6	n.d.	Bricks (Mud deposit and old furnaces)	9442	288
Ground floor: girder rust	13.7	n.d.	Reinforced concrete (New driers)	14.3	<0.1
Ground floor: wood table in the Garibaldi well cockpit	19.8	n.d.	Dust (New driers)	1100	85.7
Ground floor: door rust of the Garibaldi well cockpit	479	n.d.	Concrete (New driers)	69.6	0.9
Ground floor: mining truck rust	30.6	n.d.	Concrete (from the silo area in the New driers)	3160	185
Ground floor: rocky retaining wall	1.2	<0.1	Dust (from the silo area in the New driers)	13,680	1020
EDIFICE 10			Tuff bricks (New driers)	31.9	1.5
Ground floor (room A): painting	50.6	<0.1	Mineral wool (New driers)	58.7	2.8
Ground floor (room A): painting	21.2	n.d.	Mineral wool (New driers)	63.4	1.4
Ground floor (room A): painting	40.7	n.d.	Concrete (New driers)	23.6	0.2
EDIFICE 11			Concrete close to the transporting belt (New driers)	76.8	9.8
Ground floor (room A): painting	281	<0.1	Concrete (support of the New driers)	90.9	0.2
Rails outside Edifice 10: rust	42.0	n.d.	Concrete (condensation pools, Nesa)	64.4	1.4
EDIFICE 25			Crystalline isotactic polymer (Moplen) condensers (Nesa furnace)	17.9	7.7
Ground floor (room B): tuff wall (0–2 mm)	159	1.0	Mineral wool (Nesa furnace, first floor)	420	1.1
Ground floor (room B): tuff wall (inner part)	6.4	n.d.	Mineral wool (Nesa furnace, third floor)	336	833
Ground floor (room B): tuff wall (outer part)	21.8	12.0	Rust (Nesa furnace)	3390	351
Ground floor (room A): tuff wall (0–2 mm)	8.2	26.4	Reinforced concrete (Condensers at the Nesa furnace)	46,580	4470
Ground floor (room B): tuff brick at 10 cm depth	6.2	0.3	Mining material on the belt (conveyor belt tower)	435	3.2
Ground floor (room B): tuff brick at surface	19.7	0.8	Bricks (conveyor belt tower)	267	2550
Ground floor (room C): concrete (surface)	469	52.6	Concrete (Gould furnaces edifice)	1045	1470
Ground floor (room D): concrete (inner pat)	2.2	n.d.	Concrete (condensation pools, Gould furnaces edifice)	257	14.7
Ground floor (room D): concrete (outer part)	6.7	n.d.	Concrete close to the silos (Gould furnaces edifice)	109	6.8
Ground floor (room D): concrete	80.9	1.2	Plaster (Gould furnaces edifice)	353	34.4
EDIFICE 26			Concrete (close to the calcine deposit; Gould furnaces edifice)	76.1	20.2
Ground floor (room E): painting	122	37.6	Rust (Gould furnaces)	2490	717
Ground floor (room E): painting	81.9	n.d.	Brick (Gould furnace condensers)	6830	1690
Ground floor (room E): plaster	18.4	n.d.	Concrete (exhaust pipe; Gould furnaces edifice)	186	1.3
Ground floor (room F): painting	89.2	0.6	Bricks (liquid mercury bottling area)	4.8	10
Ground floor (room F): plaster	19.9	1.0	Lime (liquid mercury bottling area)	181	105
Main facade	42.8	n.d.	Plaster (liquid mercury bottling area)	210	392
EDIFICE 30			Plaster (liquid mercury bottling area)	325	0.66
First floor (room B): plaster	10.8	<0.1	Crystalline isotactic polymer (Moplen) condensers (Gould furnaces)	3020	660
First floor (room B): painting	25.8	<0.1	Roof tile (Gould furnaces edifice)	4.1	1.3
Roof tile	2.6	<0.1	Roof tile (Gould furnaces edifice)	485	140
			Soil in front of the Gould furnaces 1	73,670	6640
			Soil in front of the Gould furnaces 2	51,770	260

## Supplementary Materials

The following are available online at www.mdpi.com/1660-4601/14/4/425/s1, Supplementary Material 1 (SM 1), Supplementary Material 2 (SM 2).
